# Revision of a Failed Primary Total Hip Arthroplasty following Excessive Reaming with a Medial Cup Protrusion

**DOI:** 10.3390/medicina58091254

**Published:** 2022-09-10

**Authors:** Silviya Ivanova, Nicolas Vuillemin, Onur Hapa, Klaus A. Siebenrock, Marius J. B. Keel, Theodoros H. Tosounidis, Johannes D. Bastian

**Affiliations:** 1 Department of Orthopaedic Surgery and Traumatology, Inselspital, Bern University Hospital, University of Bern, 3010 Bern, Switzerland; 2 Department of Orthopaedics and Traumatology, Faculty of Medicine Dokuz Eylül University, Izmir 35330, Turkey; 3 Department of Orthopaedic Surgery, Medical School, University of Crete, University Hospital, 71003 Heraklion, Crete, Greece

**Keywords:** acetabular component, medial protrusion, acetabular fracture, total hip arthroplasty, Ganz reinforcement ring

## Abstract

*Background and Objectives*: Atraumatic intrapelvic protrusion of the acetabular component following excessive reaming of the acetabulum with a far medial positioning of the cup is a rare, but serious complication of a total hip arthroplasty (THA). This study analyzes the factors contributing to this uncommon complication and presents the outcome after the revision surgery using the Ganz reinforcement ring combined with a bone graft and plating of the posterior column and/or screws for the anterior column. *Materials and Methods*: A retrospective case series study with seven patients (four males, mean age 76 ± 10 years (60–86)) that underwent a revision THA within 24 ± 17 days (5–60) after an atraumatic periprosthetic acetabular fracture with a medial cup protrusion was performed. All fractures were reconstructed with a Ganz reinforcement ring and bone graft with a mean follow-up of 1.7 ± 1.7 years (0.5–5). Radiographs were evaluated for the following: (i) cup positioning immediately after the primary THA and the revision surgery, (ii) cup migration in the follow-up, and (iii) fracture healing. *Results*: The position of the acetabular component as assessed on the postoperative radiographs after the index surgery and before the complete medial cup protrusion showed a cup placement beyond the ilioischial line indicative of a fracture of the medial wall. The revision surgery with the reconstruction of the medial wall with a Ganz reinforcement ring combined with a bone graft restored in the presented cases the center of rotation in the horizontal direction with a statistical significance (*p* < 0.05). During the follow-up, there was no aseptic loosening with the relevant cup migration or significant change in the position of the acetabular cup at the final follow-up (*p* > 0.05) after the revision. All seven fractures and bone grafts realized a bone union until the latest follow-up. *Conclusions*: Following excessive reaming, the acetabular component was placed too far medially and resulted in an intrapelvic cup protrusion. An unstable cup following a fracture of the medial wall was evident on the immediate postoperative radiographs. In the case of the medial wall perforation with an intrapelvic cup protrusion after the primary THA, the reconstruction with a Ganz reinforcement ring was a successful treatment option resulting in the fracture healing and a stable cup positioning. Surgeons should be aware of that rare and probably underreported complication and restore the anatomic center of rotation by treating the defect intraoperatively.

## 1. Introduction

Total hip arthroplasty (THA) is a highly successful operation with excellent long-term results and a relatively low risk of complications [1]. The classic technique of a THA as described by Charnley [2] and Mueller [3] includes the medialization of the acetabular cup to the medial acetabular wall. Radiographically, the cup should not be placed medially to the ilioischial line (Kohler’s line). Excessive reaming with a resultant bone defect in the medial wall of the acetabulum and the far medial positioning of the cup, beyond the ilioischial line, can lead to a secondary intrapelvic cup migration [4] in the early weeks after surgery.

The incidence of periprosthetic acetabular fractures during the primary THA is increasing due to the use of cementless implants [5,6] and ranges from 0.4% intraoperatively [6,7] to 8.4% detected postoperatively with a routine CT scan [8]. According to some authors, the radiographic evidence of a medial breach of the acetabulum occurs in 25% of the patients after the primary THA [4]. It has been reported that there is no correlation between the medial wall fracture with an increased risk for a secondary dislocation or progression of the cup migration after full weight-bearing [4]. Nevertheless, we noticed some cases of an intrapelvic medial cup protrusion following a far medial cup positioning after the primary total hip arthroplasty in older individuals performed in other hospitals. There is still no consensus in the contemporary literature, with regards to the management of a non-displaced intraoperative acetabular fracture with many surgeons advocating for the medial wall reconstruction, whilst others support the implementation of an adjusted rehabilitation protocol [9,10,11,12,13]. The constructs utilizing antiprotrusion cages (Burch–Schneider) and reinforcement rings (Müller and Ganz reinforcement rings) along with a sufficient amount of bone graft are used to achieve an osseous consolidation and long-term stability [14,15,16]. Likewise, custom-made triflange sockets and modular trabecular titanium or tantalum implants (Trabecular MetalTM, Zimmer, Warsaw, IN, USA) can provide a ¨non-biological¨ solution by bridging the acetabular defect with osseointegrable implants [16]. 

The purpose of this study was to describe our management of a cohort of patients with this very rare injury of a medial intrapelvic protrusion of the cup following excessive reaming from the cup placement. Accordingly, this study analyses the factors contributing to this uncommon complication and presents the outcome after the revision surgery using the Ganz reinforcement ring combined with a bone graft and plating of the posterior column and/or screws for the anterior column. 

## 2. Methods and Patients

### 2.1. Patient Selection

This retrospective cohort study was conducted according to the guidelines of the Declaration of Helsinki and did not require ethical approval (in consent with the local institutional ethics committee of Kantonale Ethikkommission Bern, Switzerland, BASEC-Nr: Req-2022-00275). We retrospectively reviewed our hospital operative records between January 2014 and December 2021. The inclusion criteria were: (1) the age of patients ≥60 years, (2) an atraumatic periprosthetic fracture with a medial cup protrusion within eight weeks after the primary THA, (3) a revision with a reinforcement Ganz ring and/or bone graft and/or posterior column plate and/or anterior column screw, (4) a CT scan before the revision surgery available, (5) admitted for the first revision to our center, and (6) postoperative follow-up ≥6 months.

The medical charts of all patients were reviewed for patient demographics (age, gender, obesity (BMI), the presence of a coxa profuda or an acetabular protrusion, osteoporosis (by use of the canal bone ratio, CBR [17])), data for the primary index hip arthroplasty (surgical approach, cup/shaft design, remarked intraoperative acetabular fracture, postoperative weight-bearing) and for the revision surgery (fracture pattern, surgical approach, implants), postoperative complications according to Sink et al. [18], and for the outcome in the last follow-up. 

We identified seven (three female and four male) patients meeting the aforementioned inclusion criteria. The average age of this cohort of patients was 76 ± 10 years (range: 60–86) at the time of surgery with mean follow-up of 1.7 ± 1.7 years (range: 0.5–5). The mean BMI was 25 ± 4 kg/m^2^ (range: 19–33). Osteoporosis was diagnosed previously in only one patient, however five (71%) of all of the patients showed a positive CBR- Index [19]. All periprosthetic acetabular fractures with an intrapelvic cup protrusion occurred 24 ± 17 days (range: 5–60) after the index surgery. In Table 1 the above mentioned baseline characteristics are presented. 

### 2.2. Clinical and Radiographic Assessment

The clinical and radiological follow-up visits were planned at six weeks, three months, six months, one year after the revision operation, and annually thereafter. In the clinical follow up, the patients were assessed for the presence of a painful joint, the postoperative weight-bearing status (full vs. partial weight-bearing), and postoperative complications. In our department, a radiographic assessment is typically performed in a standardized manner which includes an anteroposterior (AP), iliac, and obturator oblique pelvic radiographs, and a cross-table radiograph of the hip [20]. For the preoperative planning, a CT scan with a metal artifact suppression is performed routinely. The fracture pattern was described using two classifications according to the AAOS [21] and Letournel [22], and for the purpose of the analysis these were divided into those involving the anteromedial elements (anterior wall, anterior column, and medial wall), posterior elements (posterior wall, posterior column, or posterior column with posterior wall) or both (transverse, transverse with a posterior wall, T-type, an anterior column with posterior hemitransverse, or both columns). The classification was performed based on the preoperative CT scans and using the operative reports. The radiographic parameters evaluated were:

(i) cup positioning directly after the primary THA and after the revision surgery, (ii) cup migration at the follow-up, and (iii) fracture healing.

#### 2.2.1. Cup Positioning

**Figure 1:**
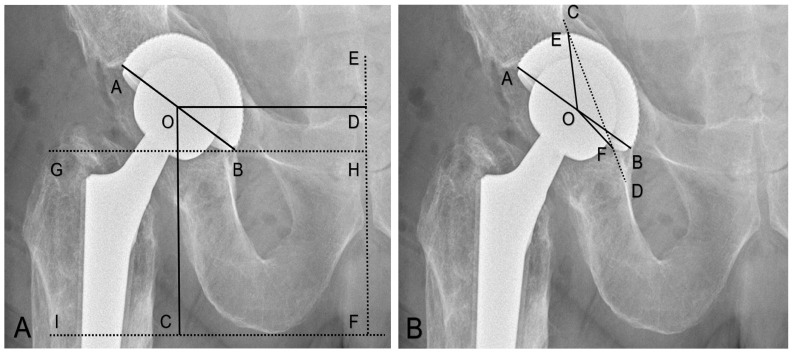
**(A**). O as the center of the femoral head, OD as the horizontal distance from the horizontal center of rotation (H-COR) to the EF midline (perpendicular line to the IF ischial tuberosity line positioned on the symphysis). OC as vertical center of rotation (V-COR) was defined as the vertical distance from the center of the femoral head to the IF ischial tuberosity line [24]. ∠ABG° as inclination was defined as the angle between the parallel line to the ischial tuberosity line and the plane of the opening of the acetabular component [23]. (**B**). Rate of the medial protrusion (∠EOF°/180°) × 100% [26]. O as the center of the femoral head. AB as the diameter of the cup. CD as the ilioischial line. Point E and point F are the intersections between the medial edge of the acetabular component and the ilioischial line.

Firstly, the position of the hip center of rotation, the inclination, and anteversion of the acetabular component on the pelvic radiograph were measured as follows:

The horizontal center of rotation (H-COR) was defined by measuring the horizontal distance from the center of the femoral head to the midline (a line perpendicular to a tangent line connecting both ischial tuberosities (ischial tuberosity line)) positioned on the symphysis [23], (Figure 1A). We defined the ΔH-COR as the difference between the H-COR on the operated hip and the H-COR on the contralateral native hip joints. 

The vertical center of rotation (V-COR) was defined as the vertical distance from the center of the femoral head perpendicular to the ischial tuberosity line [24], (Figure 1A). We defined the ΔV-COR as the difference between the V-COR on the operated hip and the V-COR on the contralateral native hip joints.

The inclination was defined as the angle between the inter-teardrop line or the ischial tuberosity line and the plane of the opening of the acetabular component [23], (Figure 1A).

The anteversion was defined as the angle between the line touching the opening surface of the acetabular component and a line perpendicularly drawn on the table on the cross-table axial radiographs [25].

Secondly, the proportion of the cup protrusion using the following parameters was determined:

The rate of medial protrusion was defined as the ratio of the degree of the cup medialization beyond the Kohler’s line and 180°: (∠EOF°/180°) × 100% [26,27], (Figure 1B).

**Figure 2:**
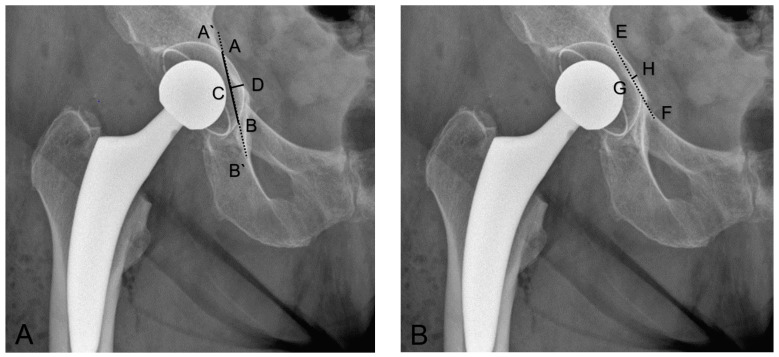
Radiographic measurements of the medial cup protrusion. (**A**). AB length of the overlap tangent was defined as the distance between the two crossings of A′B′ the ilioischial line and the cup; CD ilioischial overlap as the distance between the ilioischial line and the tangent of the acetabular cup medial. (**B**). GH iliopectineal distance as the minimal distance between the iliopectineal line (EF) and the cup [4].

The ilioischial overlap was defined as the minimal distance between the ilioischial line and a parallel line tangential to the acetabular cup [4], (Figure 2A).

The length of the overlap tangent was defined as the distance between the two crossings of the ilioischial line and the cup [4], (Figure 2A). 

The iliopectineal distance was defined as the minimal distance between the iliopectineal line and the cup. In the case of medial protrusion through the iliopectineal line, this value was negative [4], (Figure 2B).

#### 2.2.2. Cup Migration at Follow-up 

The measurements in the last radiographic follow-up (iliopectineal line, H-COR, V-COR, inclination, and anteversion) were collated to the postoperative radiographs and reported as Delta (Δ), aiming to detect any cup migration in the follow-up. An intrapelvic medial protrusion of the cup was defined as a secondary dislocation of the cup beyond the ilioischial line due to the cavitary or both cavitary and segmental fractures (AAOS type II or type III). An acetabular component was probably or definitely loosened if the migration or a change in the position of the cage or of the socket had occurred or broken fixation screws were present [28]. 

#### 2.2.3. Fracture Healing

The periprosthetic fracture was considered to be definitely healed if the bridging callus or trabecular bone was visible across the site of the fracture. The fracture was defined as non-united if a fracture line was still visible at the six-month follow-up visit or if there were other signs of failure of fracture-healing, such as a failure of the hardware or displacement of the fracture [28]. A satisfactory result was obtained if no revision on the acetabulum was required, the acetabular component was stable without any migration, the fractures healed, and if no severe pain was reported [28]. The primary endpoint of the follow-up was clinical (established full weight-bearing without any symptoms) and/or radiographic evidence for the fracture consolidation. The secondary endpoint was the need for a re-revision of the acetabular component due to the migration of the cup in the radiological control examinations.

### 2.3. Surgical Procedure

All of the primary THA surgeries were performed at an outside institution. The patients were referred to our department for the revision surgery after the radiological diagnosis of an intrapelvic cup protrusion. The indications for a total hip arthroplasty (THA) were a femoral neck fracture in one patient, primary osteoarthritis in three patients, secondary osteoarthritis after an intramedullary nailing of the pertrochanteric femoral fracture in two, and after hemiarthroplasty in one patient, respectively. A coxa profunda, defined as the location of the acetabular fossa medial to the ilioischial line on an antero-posterior radiograph [29] was noted in five of the seven patients (71%). According to the intraoperative surgical reports, reaming was performed in all seven cases without any fluoroscopic imaging control. The medial wall was violated in two cases and the medial defect was filled with cancellous bone autografts harvested from the resected femoral head in one patient. A satisfactory intraoperative cup stability was reported in all cases. The postoperative rehabilitation protocol consisted of a gradual progression to full weight-bearing in four patients and partial weight-bearing in the remaining three. 

The revision surgery in our department was performed by senior surgeons. The reaming was performed under fluoroscopic imaging. Depending upon the fracture pattern, the posterior column was fixed using reconstruction plates. If necessary, the anterior column osteosynthesis was stabilized by an anterior screw. A Ganz reinforcement ring combined with a structural or morselized bone graft in order to address bone loss was used in all cases. The bone grafts were obtained from fresh frozen head allografts stored at −80 °C before use [16]. The Ganz reinforcement ring (Sulzer Medica, Winterthur, Switzerland) with an additional inferior hook under the teardrop buttress the anterior and posterior walls, the acetabular fossa, and the dome. The ring was fixed against the area of the best bone stock with screws, which allows for the protection of the impacted bone graft and the optimal positioning and insertion of a cemented polyethylene cup [30].

### 2.4. Statistical Analysis

The continuous data are presented as frequencies (*n*) with mean values ± standard deviation (SD) and range in parenthesis. The non-parametric Wilcoxon test assessed the differences among the radiographic data. The level of significance was set at a *p*-value of *p* < 0.05. GraphPad Prism (Version 9.0, GraphPad Software, San Diego, CA, USA) was used for statistical analysis.

## 3. Results

### 3.1. Clinical Outcomes

**Figure 3:**
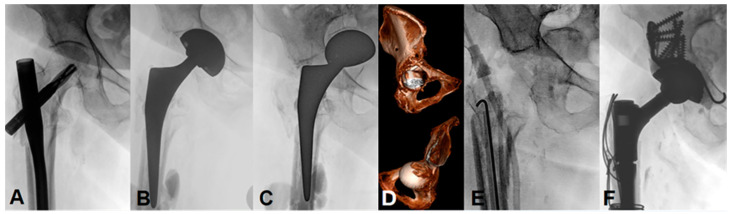
Anteroposterior radiographs and computed tomography (CT) images of an 80-year-old woman with (**A**) a symptomatic non-union of a pertrochanteric fracture on the right side, ten months after the osteosynthesis with a femoral nail. (**B**) Postoperative radiographic view two days after a THA with an interruption of the ilioischial and iliopectineal lines. (**C**) Atraumatic acetabular fracture after partial weight-bearing four weeks postoperative with a complete intrapelvic protrusion of the acetabular component. (**D**) A corresponding CT scan was acquired for evaluation of the fracture pattern (AAOS IIA) and the preoperative planning. (**E**) Two-stage revision with the removal of the femoral and acetabular components and the placement of an antibiotic-cement spacer due to the concomitant diagnosis of chronic prosthetic joint infection. (**F**) Reconstruction of the acetabulum using a dual mobility acetabular component, a structural and morselized bone graft, and a Ganz reinforcement ring, six months after the first stage of the revision.

**Figure 4:**
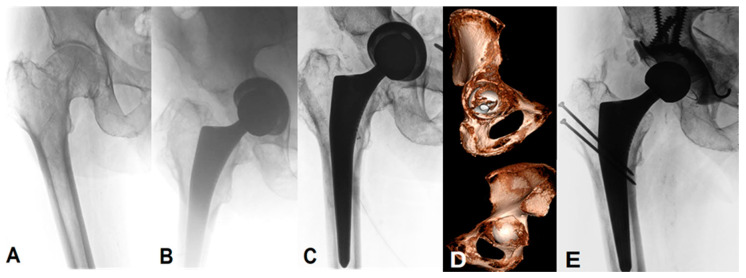
Anteroposterior radiographs and computed tomography images of an 82-year-old man with (**A**) with a symptomatic primary coxarthrosis on the right side. (**B**) Intraoperative anteroposterior view during the primary THA with an interruption of the ilioischial and iliopectineal lines. (**C**) Atraumatic progression of the acetabular fracture with an intrapelvic protrusion of the acetabular component, one day postoperative. (**D**) CT scan was performed for evaluation of the fracture pattern and the preoperative planning, showing a AAOS type IIB fracture by the presence of an extensive central defect. (**E**) Postoperative radiograph after the revision with a trochanteric flip osteotomy, a change of the acetabular component and the use of a Ganz reinforcement ring, and a structural and morselized bone allograft.

Three patients had cavitary defects (AAOS type II), three had a segmental and cavitary fracture (AAOS type III) and one suffered a pelvic discontinuity (AAOS type IV). For the revision of the unstable cup, a Ganz reinforcement ring and fracture fixation (with an anterior column screw and/or posterior column plates) was performed. One patient underwent a two-stage revision with the initial placement of an antibiotic-loaded cement spacer and a definitive acetabular reconstruction with a structural and morselized graft, the Ganz reinforcement ring, and the dual mobility acetabular component six months later (Figure 3). In relation to the primary outcome, all patients were able to practice full weight-bearing at the final follow-up (e.g., Figure 4), one patient walked with one stick and one patient with the support of two people due to preexisting chronic lumbar pain and other medical comorbidities. According to the criteria of Berry et al. [28], a satisfactory result was observed in all seven patients.

Following the revision surgery, five patients had minor grade II postoperative complications, according to Sink et al. [18]. In two patients, a re-revision unrelated to the acetabular cup was necessary: One trochanteric reattachment with a wire cerclage was performed two months postoperatively in one patient due to a secondary trochanteric avulsion. One patient sustained a postoperative hip dislocation due to disassembly of the modular femoral stem and therefore required a revision with a proximal femoral component exchange. No deep infection or sciatic nerve injuries were observed during the follow-up period. No acetabular reconstruction was noted. All of the fractures were considered to have completely healed until the latest follow-up. 

### 3.2. Radiographic Outcomes

#### 3.2.1. Cup Positioning directly after the primary THA

In the initial postoperative radiographs before the complete medial cup protrusion, the mean ilioischial overlap and the iliopectineal distance was 11 ± 3.8 mm (range: 3.5–19.8) and −3.36 ± 3.15 mm (range: −10.5–0), respectively. The length of the overlap was 43 ± 9.9 mm (range: 26.8–56.1). The rate of the medial protrusion comprised 50.6 ± 13.7% (range: 27–70). The initial cup mean inclination and anteversion were 40.9 ± 10.6° (range: 25–55) and 22.1 ± 10.8° (range: 13–45), respectively. The ΔH-COR was with a mean rate of 20 ± 7.9 mm (range: 8–35) more medial than on the contralateral native hip joint. The ΔV-COR was reported to be 7.7 ± 4.7 mm (range: 0–16) more cranial than contralateral (Table 2).

#### 3.2.2. Cup Migration in the Follow-up 

Comparing the measured parameters (iliopectineal distance, H-COR, V-COR, inclination, and anteversion) on the immediate postoperative internal radiographs and on those taken during the last follow-up, there was no aseptic loosening with a relevant cup migration or significant change in the cup position at the final follow-up (*p*-value from 0.062 to >0.333) (Table 3).

#### 3.2.3. Fracture Healing

According to the criteria of Berry et al. [28] all seven fractures and/or bone grafts realized a consolidation or bone union, respectively, until the latest follow-up. The radiographic assessment values are shown in Table 3. 

## 4. Discussion

The incidence of intraoperative and early postoperative periprosthetic acetabular fractures after the primary THA is increasing according to the use of cementless implants [5,6]. Our institution is a tertiary referral center for revision hip arthroplasty and it has recently come to our attention that there are a number of cases of atraumatic intrapelvic cup penetration through the quadrilateral plate after a primary total hip arthroplasty in older patients in the early postoperative course. Due to a very low incidence, this fracture pattern is described only in two case reports [31,32], hence, there is a lack of information for their etiology and treatment. 

The aim of our study was to present an unusual complication in the primary THA and to supply a feasible intra- and/or postoperative problem-solving strategy, providing a proposal for the successful prevention and management. To address this issue, we investigated the clinical and radiological parameters related to this complication. Therefore, we evaluated radiographically the position of the acetabular component in order to elucidate an early postoperative migration of the cup after the primary THA; to ensure the presented revision surgery was successful, the radiographic assessments were repeated after the revision THA as follows: Firstly, we measured the center of the hip rotation (H- and V-COR) as an important, established reference point for the optimal position of the acetabular component in both medial-lateral and cranial-caudal directions and compared the values with the contralateral native side [24]. Therefore, we measured the inclination and anteversion of the cup in a standard manner as described previously [23,25]. Secondly, we measured the rate of the medial cup protrusion according to the methods introduced by Dorr et al. [33] to determine the cup coverage during the primary THA in patients with developmental hip dysplasia [26,27]. Furthermore, we determined the ilioischial overlap, the length of the overlap tangent, and the iliopectineal distance, according to Mandelli et al. [4]. The ilioischial overlap was reported as a reliable parameter to measure the position and migration of the acetabular cup; furthermore, the overlap tangent length and the iliopectineal distance showed an excellent intra-observer reliability as well [4]. Finally, we compared the measured parameters to those in the last follow-up, to report any cup migration and to prove the fracture healing. 

In contrast with previous studies [4] we observed a clearly shorter iliopectineal distance with a negative value, which is indicative that the acetabular component was already intraoperative or immediately postoperative beyond the iliopectineal line. Our visibly longer ilioischial overlap as a sign of the distance beyond the ilioischial line and the longer overlap tangent length, respectively, indicates a fracture of the medial wall with a cup protrusion. We reported in these cases 100% of intrapelvic cup penetration directly postoperative or during routine activities, early postoperatively. Although it is impossible to extrapolate our results to a broader population, we observed the same tendency for far deeper reaming in all of our cases. Therefore, if reaming is performed beyond Kohler’s line, an image intensifier should be used and great attention should be paid to presence of a coxa profunda, and also whether or not the involvement of the iliopectineal line is observed.

Reaming to the acetabular floor can lead to a significant displacement of the COR medially and superiorly [23]. We reported rates of directly postoperative medial cup protrusions of 50.6 ± 13.7% (range: 27–70), which can explain, to some extent, the medial intrapelvic cup displacement in our patient cohort. Previous studies emphasized that the rate of medial protrusion defined as the percentage of the cup beyond the ilioischial line should be less than 45% for THA in patients with developmental hip dysplasia [33]. However, Kim et al. [34] recommended a protrusion rate within 50–60% and Zha et al. reported a protrusion rate of <60% [27] in order to obtain excellent clinical and radiographic midterm results. There is no consensus on the cup position medially [26], however, according to these results, we tend towards a rate of medial protrusion of no more than 50% if a medial breach cannot be precluded.

Even though that these data give us a significant amount of information, we cannot extrapolate results from the patients with an acetabular reconstruction for hip dysplasia to the patients undergoing a primary THA for osteoarthritis in general. The H-COR after the primary THA in our cohort was more medially and the V-COR was more superiorly placed than the contralateral side. One of the main goals in the THA is the placement of the acetabular component on the anatomical hip center of rotation. In order to preserve the acetabular bone stock and avoid any soft-tissue impingement, dislocation, impaired kinematics of the hip, and long-term loosening due to displacement of the COR, it has been suggested that the COR should be restored to <3 mm superiorly and <5 mm medially to the COR in a normal, healthy hip [23]. Miles and McNamee et al. demonstrated that the medial displacement of the COR, as measured in our cases, resulted in increased compressive stresses on the medial wall and tensile stresses on the lateral wall of the acetabulum, which predispose to a loosening of the component [23,35]. According to this, the medial cup protrusion in our cases might be caused by excessive reaming with a too medially positioned H-COR of the cup without the reconstruction of the anatomical H-COR. The reconstruction of the medial wall with a Ganz reinforcement ring combined with a bone graft, restored in the presented cases the center of rotation in the horizontal direction. Accordingly, surgeons should be aware of excessive reaming and assess intraoperatively for a far medial cup positioning, and immediately restore the COR by treating the defect as presented here. 

Hedley et al. discussed already, in 1982, that a medial penetration during reaming did not result in a displacement or medial migration of the acetabular component in canine models, when bone paste was used to augment the medial defect producing new bone growth [36]. Additionally, Mandelli et al. [4] did not find an increased risk for a secondary cup dislocation in patients with a postoperative radiographic medial protrusion of the acetabular component beyond the ilioischial line even when full weight-bearing was applied postoperatively. This is quite reproducible, since the medial protrusion technique consisting of a controlled medial wall fracture, a medial wall osteotomy, and/or a wall penetration is an established treatment in patients with developmental dysplasia of the hip [27]. The series of methods deepens the acetabulum and insert the cup with a medial aspect beyond the ilioischial line to achieve a higher rate of cup coverage [26]. However, the deepening continues simply until it reaches the outer surface of the internal pelvic cortex and ideally does not perforate it [26]. According to the study of Mandelli et al. [4], no intraoperative repair of the medial defect was performed as long as the cup appeared to be stable under direct manipulation. However, this method is arguable, since all initial operation reports in our cases showed a stable cup after the intraoperative mechanical control, according to the theatre notes. 

None of the patients in the above mentioned studies of hip dysplasia [27], however, developed, apart from the medial wall perforation, an acetabular column or wall fracture during the operation. In our cohort, three of the patients had additional atraumatic fractures of the anterior or posterior column (AAOS type III) and one showed a pelvic discontinuity on a computed tomography. Despite this, we cannot make any statement regarding an intraoperative occult acetabular fracture. However, 86% of the patients (*n* = 6) manifested in the intraoperative or directly postoperative radiograph, a protrusion beyond the iliopectineal line. Additionally, the patients, on which the primary THA for hip dysplasia was performed, are usually younger than hip osteoarthritis patients with limited bone stock [26]. In contrast, periprosthetic acetabular fractures occur in considerably older individuals. However, it is unclear if the cup impaction into a sclerotic, osteoarthritic bone, compared with a normal bone, would affect the initial stability at the implant–bone interface [37], even if it appears obvious. 

To increase the precision and accuracy of the acetabular cup position, leg length and offset robotic and computer navigation technologies are used. Based on the currently available level one randomized controlled trials, conventional THA results in significantly shorter surgical times and a similar incidence of complications and revisions compared with robotic-assisted and computer navigated THAs [38]. Based on the missing significant benefit in the clinical outcome and decrease of postoperative complications, coupled with the increased substantial costs, the superiority of the navigated THA remains controversial [39]. However, the preoperative imaging evaluation and thorough preoperative planning is of utmost importance, in doubt the intraoperative use of a fluoroscan can be used to rule out far medial and excessive reaming. 

This study has some limitations. First, this is a retrospective study of a small number of cases of a failed primary THA due to excessive reaming, that were performed in other hospitals. A sample size of seven cases makes the study underpowered in order to determine the therapeutic strategies and to make recommendations. Nevertheless, this complication is disastrous but relatively unknown, perhaps due to underreporting or its rarity per se, as only three cases in the last 22 years have been published [31,32], to our knowledge. Second, comparing the measured values on radiographs from different studies is not completely reliable; we nevertheless collated the measurements on the radiographs taken in a standardized fashion [4]. Third, the follow-up was rather short with an average of 1.7 years since some of the patients performed their radiologic exams at their referring local hospitals after completing the acute postoperative recovery period. An excessive reaming causes loss of bone stock of both columns and decreases the initial stability of the cementless cups [27]. This results in early or late cup loosening. According to the Annual Report of the Swiss National Joint Registry (SIRIS) from 2019 [40], the aseptic loosening of the acetabular component was the second most common reason for revision between 2015 and 2018, and it amounts to up to 18.1%. However, during the initial six-week postoperative period, the cup in the primary THA for acetabular fracture can be expected to migrate by one to three mm centrally and superiorly, and then stabilize itself in the consolidating bone [41]. If the cup is displaced by more than three to five mm, it may undergo spontaneous loosening and misalignment, necessitating a revision [41]. The latest radiographs of our patients showed a good stability of the acetabular cup with the cup migration of Δ1.6 mm between the immediate and the last postoperative radiographs. The acetabular fractures were thus far all consolidated. Therefore, we would not expect a relevant further cup migration requiring a revision in a longer follow-up period.

The rare nature of this complication makes it challenging to provide prospective randomized studies. One strength of this study lies first in the number of atraumatic intrapelvic cup protrusions reported, which, according to our literature research, presents the largest cohort. Secondly, our treatment strategy showed a satisfactory clinical and very good radiograph outcomes with the consolidation of all fractures after the revision surgery. Finally, we noticed that an intrapelvic protrusion of the acetabular component is rarely observed or reported, with only two articles and a total of three cases reported. Additionally, we found in our hospital records, seven cases that were referred to our center for a THA revision surgery due to that complication and managed using the Ganz reinforcement ring and a satisfying outcome was reported during the follow-up period. Furthermore, no significant migration of the cup was found on the pelvic radiographs during the follow-up.

## 5. Conclusions

Secondary intrapelvic protrusions of the acetabular component following excessive reaming of the acetabulum with far medial positioning of the cup with or without an intraoperative periprosthetic fracture is rare or underreported, but it is a disastrous complication of the THA. An intraoperative image intensifier should be used if navigation and robotics are unavailable to control the cup positioning in all cases with poor bone stock, especially when reaming is at risk to reach beyond the Kohler’s teardrop and/or in the presence of a coxa profunda. The medialization of the H-COR of >5 mm in comparison with a normal COR and the positioning of the acetabular component beyond the iliopectineal line with a consecutive medial cup protrusion of >45–50% should be avoided. In case of a medial wall perforation with an intrapelvic cup protrusion, the reconstruction with a Ganz reinforcement ring combined with a bone graft and plating of the posterior column and/or screws for the anterior column, if necessary, is a safe and successful treatment.

## Figures and Tables

**Table 1:** Baseline characteristics of the study population.

No.	Age	Gender	BMI (kg/m^2^)	Coxa Profunda	CBR-Index	Fracture IS	Follow-up (m)	Fracture Type AAOS	AC Involvement	PC Involvement
1	80	f	33	+	0.52	-	8	IIA	-	-
2	86	m	26	-	0.47	-	12	III	+	-
3	84	f	19	+	0.53	-	8	III	-	+
4	82	m	25	+	0.43	-	7	IIB	-	-
5	78	f	23	+	0.49	-	63	III	+	-
6	62	m	28	-	0.51	+	6	IIB	-	-
7	60	m	24	+	0.43	+	37	IV	+	+

BMI body mass index; CBR-index canal bone ratio- index; Fracture IS was noticed during the index surgical operation; Follow-up in m months; Classification according to the AAOS (American Academy of Orthopedic Surgeons); AC anterior column involvement, PC posterior column. +: Presence; -: Absence

**Table 2:** Radiological measurements after the index operation.

No.	Ilioischial Overlap (mm)	Length of Overlap (mm)	Iliopectineal Distance (mm)	Rate of Medial Protrusion (%)	ΔH-COR (mm)	ΔV-COR (mm)	Inclination (°)	Anteversion (°)
1	14.0	51.4	−3.0	65	17	7	55	16
2	5.3	37.9	0.0	40	26	4	42	45
3	3.5	26.8	−1.0	27	17	7	25	26
4	19.8	56.1	−10.5	70	35	10	45	13
5	6.1	35.5	−2.1	50	21	0	53	27
6	10.0	41.3	−3.4	46	16	16	28	13
7	18.6	52.6	−3.5	56	8	10	38	15

Ilioischial overlap defined as the distance between the ilioischial line and a tangent of the acetabular cup medial; length of overlap as the distance between the two crossings of the ilioischial line and the cup; iliopectineal distance as the minimal distance between the iliopectineal line and the cup; Rate of medial protrusion in % as the ratio of the degree of cup medialization beyond the Kohler’s line and 180°: (∠EOF/180°) × 100 (Figure 1); H-COR as the horizontal center of rotation, ΔH-COR as the difference between the H-COR on both sides; V-COR as the vertical center of rotation, ΔV-COR as the difference between the V-COR on both sides.

**Table 3:** Radiographic parameters and outcomes.

Parameter	PI	PR	*p*-Value (PI vs. PR)	FU	ΔFU-PR	*p*-Value (PR vs. FU)
Iliopectineal distance (mm)	−3.36 ± 3.15 (−10.5–0)	11 ± 7.2 (1.6–24)	0.015	13.3 ± 6.4 (7–25)	1.6 ± 2.2 (0–6)	0.062
ΔH-COR (mm)	20.0 ± 7.9 (8–35)	9.9 ± 5.2 (1–17)	0.015	10.9 ± 4.6 (6–20)	1 ± 5.7 (−8–7)	0.333
ΔV-COR (mm)	7.7 ± 4.7 (0–16)	5.7 ± 3.7 (1–11)	0.468	5 ± 3.5 (0–10)	−0.7 ± 2.5 (−5–3)	0.271
Inclination (°)	40.9 ± 10.6 (25–55)	41.6 ± 6.6 (31–54)	0.937	42.3 ± 7.3 (33–55)	0.7 ± 2.5 (−2–6)	0.312
Anteversion (°)	22.1 ± 10.8 (13–45)	16.7 ± 7.5 (9–32)	0.234	15.0 ± 7.0 (4–27)	−1.7 ± 4.5 (−10–4)	0.109

Radiographic measurements after the index operation (PI post index), after the revision operation (PR post revision) and during the last follow-up (FU). ΔFU-PR Delta as difference in the measured values between PR and FU. Results are presented as means ± standard deviation (range). H-COR as the horizontal center of rotation, ΔH-COR as the difference between the H-COR on both sides; V-COR as the vertical center of rotation, ΔV-COR as the difference between the V-COR on both sides.

## Data Availability

The data presented in this study are available on request from the corresponding author. The data are not publicly available due to privacy of patient data.
